# Evaluation of the diagnostic value of Sentinel Lymph Node in patients with gastric adenocarcinoma

**DOI:** 10.22038/AOJNMB.2023.70461.1491

**Published:** 2024

**Authors:** Ramin Sadeghi, Reza Taheri, Ali Jangjoo, Akbar Pakdel, Mohammad-Hassan Arjmand, Mohammad Reza Motiei, Bahram Memar, Mohsen Aliakbarian

**Affiliations:** 1 Nuclear Medicine Research Center, Mashhad University of Medical Sciences, Mashhad, Iran; 2 Surgical Oncology Research Center, Mashhad University of Medical Sciences, Mashhad, Iran; 3 Student Research Committee, Mashhad University of Medical Sciences, Mashhad, Iran; 4 Transplant Research Center, Clinical research institute, Mashhad University of Medical Sciences, Mashhad, Iran; 5 Department of Pathology, Emam Reza Hospital, Mashhad University of Medical Sciences, Mashhad, Iran

**Keywords:** Sentinel lymph node biopsy Gastric adenocarcinoma, Metastasis, Isosulphan blue dye

## Abstract

**Objective(s):**

Sentinel lymph node biopsy (SLNB) has been proven as a safe and efficient procedure in some cancers like breast cancer and melanoma with a reduction of complications and side effects of unnecessary lymphadenectomy in many patients. However, the diagnostic value of SLNB in gastric cancer is a point of debate. This study evaluated the diagnostic value of SLNB using radiotracer and isosulphan blue dye injection in patients with Gastric Adenocarcinomas (GA).

**Methods:**

This descriptive study was performed at Imam-Reza HOSPITAL on 39 patients diagnosed with GA with no lymphatic metastasis using two methods: the combination of radionuclide with isosulphan together (R&I) method compared with the isosulphan alone method. Lymphatic dissection was performed in all patients. The pathological results were compared between the sentinel lymph nodes (SLN) and other lymph nodes and their accordance rate was calculated.

**Results:**

In the T1 group, the sentinel lymph node biopsy detection rate was 100% for the combination of the R&I method and 60% for the isosulphan method and the false negative rate was zero. These values respectively were 88.8% and 88.8% in the T2 group with a false negative rate of 75%. In the T3 group, the values were 100% for the combination of the R&I method and 93.7% for the isosulphan method with a false negative rate of 40%. In the combination of the R&I method, the sensitivity, specificity, and positive and negative predictive values were 57.9, 100, 100, and 69.2 percent respectively.

**Conclusion:**

Based on the false negative rate (47.4%), SLNB by injection of isosulphan blue dye alone is not a diagnostic enough value for predicting lymph node metastasis in GA. Although, SLNB by combination of the R&I had better accuracy compared to the isosulphan alone, more studies with larger samples are needed to prove this result.

## Introduction

 GA is one of the most common cancers worldwide (1). As most Gastrointestinal neoplasms metastasize via the lymphatic system, detecting these lymph nodes and their resection is important in patients' survival and in reducing the recurrence rate (2, 3). 

 A valuable method for detecting malignant lymph node metastasis in patients with clinically lymph node-negative is lymphatic mapping technology and SLNB, which are approved for Breast cancer, Melanoma, and some other cancers (4-7). This method is based on the concept that if metastasis has occurred to 

the lymphatic chains, the sentinel node is the first to reach by metastasizing high confidence rate. SLNB is considered a valuable method based on lymphatic mapping and sentinel lymph biopsy, and its valuable diagnostic role has been proven in many cancers (8, 9). The detection of SLN in patients is considered using a radionuclide and isosulphan blue dye (10).

 Lymphadenectomy is a widely accepted procedure to reduce the risk of metastasis in GA. Gastrectomy with lymphadenectomy is the standard surgical method for patients with GA in Japan and many other countries (11, 12). 

 Many studies have shown that total lymphadenectomy in patients with GA without lymph node metastasis has not led to better survival and increased side effects (13), so SLNB as a relatively complication-free detective method could reduce unnecessary incisions and replacement instead of unnecessary lymph-adenectomy at first (14). Moreover, Accurate staging in Gastric cancer requires the evaluation of at least 15 lymph nodes which is very costly and time-consuming (15). 

 Therefore, SLNB could improve staging, reduce costs, and save time by concentrating on one or a limited number of lymph nodes. SLNB is a valuable method based on lymphatic mapping and sentinel lymph biopsy. Its appreciated diagnostic role has been proven in many cancers. In one study, Cozagglio and colleagues have shown that SLNB using isosulphan blue is technically possible but has low sensitivity and further studies with a large accrual are needed (16). Hayashi et al. reported the detection rate of SLNB by using a dye alone 90%, a radioactive substance alone 90%, and then using both (100%) and reported the false negative rates (17). In a standard surgery D2 gastrectomy, lymphatic dissection around the sympathetic and other arteries must be performed, which is time-consuming and requires high experience, and increases the possibility of complications. If SLNB approved to be reliable and no need for routine dissection, the time and complications of this surgery will be reduced (3, 18, 19).

 This study aims to evaluate the diagnostic value of SLNB in patients with GA using the isosulphan alone and the combination of radionuclide and isosulphan. If approved, this method could reduce unnecessary incisions and side effects and help the better pathologic staging of the disease.

## Methods

***Patients***

 This study enrolled 39 patients with GA referred to the Department of Surgical Oncology of Imam Reza Hospital in Mashhad between December 2019 and July 2021 to detect SNLs. Diagnostic methods include chest X-ray, ultrasonography, or abdominal CT scan. Patients without pathological proof of stomach cancer, involvement of lymphatic metastasis during the surgery, metastatic or unresectable cancer, and neoadjuvant chemotherapy were excluded from the study. Informed consent was obtained for all patients before recruitment into the study.

***Procedure***

 Before performing any dissection, a radioactive substance (Tc-99m antimony sulfide colloid in 0.5 ml saline) was injected subserosally in four points around the tumor at the beginning of the operation, which was equivalent to 2 millicuries. Then the blue colored substance (Guerbet Patent Blue V Sodium 2.5% diluted with 2 ml of normal saline) was injected subserosally in four different places around the stomach tumor. 10 minutes after the dye was injected, its diffusion was investigated.

 After the injection of the radionuclide material, omentectomy was started with minimal lymphatic manipulation, and in the next step, the duodenum stoma was separated and closed. After performing these steps, the lymph nodes with the highest absorption rate of radioactive substance and all lymph nodes with the absorption rate of radioactive substance greater than 10% of the hottest node and all the nodes stained by patent blue were removed and the anatomical location of all removed lymph nodes was marked. Gastric specimens and lymph nodes were assessed for pathologic evaluation. All patients' information and pathological characteristics in the data were coded.

 Then, routine radical gastrectomy was performed in all patients. Before ending the operation, the entire abdominal cavity was examined with a manual gamma probe to ensure the absence of residual radioactivity.

***Statistics***

 Data were analyzed using Statistical Package for Social Sciences (SPSS) version 23 (SPSS Inc., Chicago, IL, USA). P-values of ±standard deviations were analyzed using the Students’-test. The Fisher's test was used to analyze categorical data. The detection rate, sensitivity, and specificity, positive and negative predictive values for Sentinel lymph node biopsy were calculated.

## Results

 39 patients were registered in our study, but respectively 2 and 5 of them exclude from the study in the combination of the R & I method and isosulphan method because there was no detection of SLN. The Characteristics of patients include age, sex, and some characteristics of tumors are shown in Table 1. The average patient age was 61.3, with 7 women and 32 men. 5 patients had T1 disease, 18 patients had T2 disease, and 16 had T3 disease. The stage of lymph node involvement was N0 in 16 patients, N1 in 9, N2 in 4, and N3 in 5 patients.

**Table1:** Characteristics of the patients

	
Total number of patients	39
Age (years)	61.3 (37-79)
Sex (male/female)	32/7 (82%/18%)
Tumor location (Antrum/Fundus/Body)	26/5/8
Type of tumors (Adenocarcinoma/ GIST)	37/2 (95% / 5%)
**T stage (TNM classification)**	
T1	5 (12.8%)
T2	18 (46.2%)
T3	16 (41%)
**N stage (TNM classification)**	
N0	19 (48.7%)
N1	11 (28.2%)
N2	4 (10.3%)
N3	5 (12.8%)
SLN detected with isosulphan	34 (87.2%)
Number of SNL detected with combination of R&I	37 (95%)

GIST: Gastro Intestinal Stromal Tumor, SLN: Sentinel Lymph Node, R&I: Radionuclide and Isosulphan

Tables 2 and 5 show the pathological characteristics of SLN by a combination of radionuclide and isosulphan. Between 37 patients, 11 patients had positive SLN (SLN+). 

 After the tissue analysis, we found that all of the patients with SLN+ had metastasis to other lymph nodes. In addition, in 26 patients with SLN-, 18 patients had no other lymph node involvement (table 2). Related to the tumor stage, most patients were found with T2 and T3

cancer, and the T1 stage was reported just in 5 patients with no SLN involvement and no metastasis in other lymph nodes. Pathological characteristics of SLN by Isosulphan are summarized in tables 3 and 4. Between 34 patients, 10 patients had positive SLN (SLN+) with metastasis to other lymph nodes. Moreover, in 24 patients with SLN-, 15 patients had no other lymph node involvement (table 3). 

**Table2:** Status of SLN by a combination of R&I and other lymph nodes (LN)

	**LN+**	**LN-**	**Total**
SLN+	11	0	11
SLN-	8	18	26
Total	19	18	37

LN+: Other Lymph Node with Metastasis, LN-: Other Lymph Node without Metastasis, R&I: Radionuclide and Isosulphan,

SLN+: Sentinel Lymph Node with Metastasis, SLN-: Sentinel Lymph Node without Metastasis

**Table3:** Status of SLN by isosulphan and other lymph nodes (LN)

	**LN+**	**LN-**	**Total**
SLN+	10	0	10
SLN-	9	15	24
Total	19	15	34

LN+: Other Lymph Node with Metastasis, LN-: Other Lymph Node without Metastasis, SLN+: Sentinel Lymph Node with Metastasis, SLN-: Sentinel Lymph Node without Metastasis

 Related to the tumor stage, most patients were found with T2 and T3 cancer and just 3 patients with no SLN involvement and no metastasis in other lymph nodes were reported in the T1 stage. As a result, most of the lymph node metastasis is related to T2 and T3 (Tables 4 and 5). 

 Some analytical parameters about the accuracy of SLNB were reported in Table 6. 

 Using SLNB with a combination of radionuclide and isosulphan showed 78.3% accuracy, 57.9% sensitivity, and 100% specificity, and positive and negative predictive values were 100% and 69.2%, respectively. Also, the false negative was 42.1. The result is summarized in Table 6.

**Table4:** The relationship between SLN (using isosulphan) with T stage

**T stage**		**LN+**	**LN-**	**Total**
T1	SLN+	0	0	0
	SLN-	0	3	3
T2	SLN+	1	0	1
	SLN-	3	12	15
T3	SLN+	9	0	9
	SLN-	6	0	6
Total		19	15	34

LN+: Other Lymph Node with Metastasis, LN-: Other Lymph Node without Metastasis, SLN+: Sentinel Lymph Node with Metastasis, SLN-: Sentinel Lymph Node without Metastasis

**Table5:** The relationship between SLN (using R&I) with T stage

**T stage**		**LN+**	**LN-**	**Total**
T1	SLN+	0	0	0
	SLN-	0	5	5
T2	SLN+	1	0	1
	SLN-	3	12	15
T3	SLN+	10	0	10
	SLN-	5	1	6
Total		19	18	37

LN+: Other Lymph Node with Metastasis, LN-: Other Lymph Node without Metastasis, R&I: Radionuclide and Isosulphan,

SLN+: Sentinel Lymph Node with Metastasis, SLN-: Sentinel Lymph Node without Metastasis

**Table6:** The analytical parameters about the accuracy of SLNB

	**SLN using isosulphan**	**SNL using combination of R&I**
Accuracy	75.6	78.3
Sensitivity	52.6	57.9
Specificity	100	100
False negative	47.4	42.1
False positive	0	0
PPV	100	100
NPV	62.5	69.2

NPV: Negative Predictive Value, PPV: Positive Predictive Value

## Discussion

 The diagnostic SLN technique in gastric cancer was performed in the late 1990s (16, 20). One study by Yaguchi et al. showed that sub-serosal and sub-mucosal injections for evaluation of lymph node involvement had no significant difference (21). Regarding lymph node biopsy in Gastric cancer, Tangoku et al. have reported that lymph node involvement is close to zero when the disease is at the mucosal level but rises to 20 percent as the disease spreads to the submucosal area. Therefore, lymphadenectomy is conservatively done in mucosal Gastric cancer, but extensive dissection of lymph nodes is performed in submucosal Gastric cancer as in more advanced cancer; unless SLNB finds its place. The authors also state that due to the 15–20 percent rate of skipped metastasis in Gastric cancer, finding the sentinel lymph node could be both therapeutic and prognostic (22). 

 According to Cheng's study, the first metastasis is not always in the perigastric area and skipped metastasis is common in gastric cancer; blind evaluation of lymph nodes around the tumor is unreliable. If the pre-operative studies show submucosal invasion, it is necessary to perform extensive lymph node assessment to find sentinel lymph nodes (23, 24). Several studies have indicated that lymph node assessment should be done very carefully in Gastric cancer and melanoma for which SLNB has long been performed (25, 26). Moreover, Hayashi et al. evaluated the manifestation rate of dye substance alone, radioactive substance alone, and the two together, which were 90%, 90%, and 100 %, respectively. They have also reported their false negative rates and concluded that it is better to use both substances when assessing sentinel lymph nodes (17). According to the study reported by Schlag, although SLNB might not reduce the number of resected nodes, this method has gained much notice in improving the staging of the disease (27). In another study by Rossi et al, detection metastases in inendometrial cancer by identifying Sentinel lymph nodes with indocyanine green showed a high degree of diagnostic accuracy (18). Our study showed that SLNB using isosulphan alone could not be sufficient to diagnose lymph node involvement in Gastric cancer due to the high rate of skipped metastasis. Moreover, SNLB using a combination of isosulphan and radionuclide compared to SLNB using isosulphan alone had better diagnostic value for the detection of lymph node involvement in Gastric cancer.

## Conclusion

 According to our result comparing the accuracy, false negative, and sensitivity between SLNB using isosulphan alone and SNLB using a combination of isosulphan and radionuclide, SLNB using isosulphan could not alone be sufficient to diagnose lymph node involvement in Gastric cancer. The main challenge for the poor accuracy may be the variability of the lymphatic routes in the gastric site, consequential in a high rate of skipped metastasis. Although SNLB using a combination of isosulphan and radionuclide compared to SLNB using isosulphan alone had better diagnostic value for detection of lymph node involvement in Gastric cancer, more investigations are needed for complete information before this method can be used as a common diagnostic procedure. 

## Declaration

 Conflict of interest: the authors declare no competing interests directly related to this work.

## Competing interest

 Not applicable.

## Funding

 This study was funded by Mashhad University of Medical Science (88530).
